# Integrative gene transfer in the truffle *Tuber borchii* by *Agrobacterium tumefaciens*-mediated transformation

**DOI:** 10.1186/s13568-014-0043-x

**Published:** 2014-05-29

**Authors:** Andrea Brenna, Barbara Montanini, Eleonora Muggiano, Marco Proietto, Patrizia Filetici, Simone Ottonello, Paola Ballario

**Affiliations:** 1 Pasteur Cenci Bolognetti Foundation, c/o Department of Biology and Biotechnology “Charles Darwin”, Sapienza University, Piazzale A. Moro 5, Rome, 00185, Italy; 2 Department of Life Sciences, Biochemistry and Molecular Biology Unit, Laboratory of Functional Genomics and Protein Engineering, University of Parma, Parma, 43124, Italy; 3 Institute of Biology and Molecular Pathology, CNR, Rome, 00185, Italy

**Keywords:** Tuber spp, Truffles, Agrobacterium tumefaciens-mediated transformation, T-DNA, binary plasmid, Green fluorescent protein, Hygromycin phosphotransferase, TAIL-PCR

## Abstract

*Agrobacterium tumefaciens*-mediated transformation is a powerful tool for reverse genetics and functional genomic analysis in a wide variety of plants and fungi. *Tuber* spp*.* are ecologically important and gastronomically prized fungi (“truffles”) with a cryptic life cycle, a subterranean habitat and a symbiotic, but also facultative saprophytic lifestyle. The genome of a representative member of this group of fungi has recently been sequenced. However, because of their poor genetic tractability, including transformation, truffles have so far eluded in-depth functional genomic investigations. Here we report that *A. tumefaciens* can infect *Tuber borchii* mycelia, thereby conveying its transfer DNA with the production of stably integrated transformants. We constructed two new binary plasmids (pABr1 and pABr3) and tested them as improved transformation vectors using the green fluorescent protein as reporter gene and hygromycin phosphotransferase as selection marker. Transformants were stable for at least 12 months of in vitro culture propagation and, as revealed by TAIL- PCR analysis, integration sites appear to be heterogeneous, with a preference for repeat element-containing genome sites.

## Introduction

The ascomycete *Tuber borchii* is a hypogeous fungus (genus, *Tuber*; family Tuberaceae; order Pezizales) that establishes a beneficial mutualistic interaction (‘ectomycorrhiza’) with the roots of many tree species. *T. borchii* has also a pronounced saprobiotic capacity and can be grown in vitro (albeit quite slowly) as vegetative free-living mycelium in the absence of a plant host. This mixed symbiotic/saprophytic lifestyle (Hebe et al. [1999]), together with the lack of asexual spores amenable to in vitro culture has hampered the genetic manipulation of this fungus and obscured our understanding of its complex life cycle. *Tuber* fruitbodies (‘truffles’) lack an active system for the dispersal of spores, which are disseminated by the action of mycophagous animals (Pegler et al. [1993]). Another peculiarity of certain *Tuber* spp. is the high commercial value of their fruitbodies which are prized as gourmet food. *Tuber melanosporum* and *T. borchii* are the two most studied truffle species. Sequencing and annotation of the *T. melanosporum* genome, the first symbiotic Ascomycete to be sequenced, revealed many aspects of truffles’ biology and genetic organization, including their massive content of repeated transposable elements and their heterotallic mode of conjugation and ability to outcross through the action of two distinct mating type loci (Martin et al. [2010]). *T. borchii* is the truffle that can be more easily handled under laboratory conditions.

With the acquisition of new genome sequence data, functional genetic studies are all the more necessary to decipher gene function. Reverse genetics in *Tuber* has been hindered, however, by the absence of a well-established stable transformation system. An effective approach in this direction is represented by *Agrobacterium tumefaciens*-mediated (ATM) transformation of intact hyphae. This technique was first successfully applied to fungi 19 years ago (Bundock et al. [1995]) by exploiting the natural ability of *A. tumefaciens* to transfer a portion of its DNA to a foreign infected organism, most notably dicotyledonous plants. To date, ATM transformation is applied to the study of a variety of fungal species, including Ascomycetes, Basidiomycetes and Zygomycetes. The transfer DNA (T-DNA), located on a >200 kb tumor-inducing (Ti) plasmid, is flanked by two 25 bp direct imperfect repeats, known as left border (LB) and right border (RB) sequences, which also include the *vir* genes encoding the genetic factors required for transfer (Zupan et al. [2000]). Recent improvements of ATM transformation include the optimization of temperature and co-cultivation conditions, and the development of new selection markers (Michielse et al. [2005]). We previously described an ATM transformation procedure for *T. borchii* (Grimaldi et al. [2005]), which due to the insertion of transgenic DNA driven by the T-DNA, and consequent lack of stability, could not be effectively exploited for functional genomics studies. Building upon these earlier attempts, in the present work we improved transformation conditions through the development of two new vectors and also gained insight into the fate of the transferred T-DNA, which was found to be randomly integrated, with a preference for repeat element-containing genome sites. These results represent an important technical advancement for the molecular biological investigation of truffles, which will be instrumental to the construction of mutant strain collections.

## Materials and methods

### Strains and media

*T. borchii* Vittad. mycelia (isolate ATCC 95640) were grown and propagated in the dark at 24°C on potato-dextrose medium with agar 39 g/l (PDA: 0.2% peptone, 0.2% yeast extract, 1.8% glucose, 0.5% potato starch, 1.5% agar; Liofilchem), as described (Ambra et al. [2004]). For easy medium replacement, mycelial cultures were usually grown on sterile dialysis membranes. For liquid culture replacement, mycelial cultures were inoculated in potato-dextrose liquid medium (PDB, without agar). *A. tumefaciens* was grown and propagated in Luria- Bertani (LB) (1% tryptone, 0.5% yeast extract, 1.5% agar) solid or liquid (w/o agar) medium at 28°C in the presence of appropriate marker selection supplements. After co-cultivation, mycelia were transferred onto Evans minimal medium (1x Vogel’s salts, 1% glucose, 2 mg/l vitamin B1, 1.5% agar). The hypervirulent *Agrobacterium* strain AGL-1 [(C58 pTiBo542) recA::bla, T-region deleted Mop (+) Cb (R)] was kindly provided by Peter Romaine (Department of Plant Pathology, Pennsylvania State University, University Park, PA, USA) (Lazo et al. [1991]). Carbenicillin (Sigma-Aldrich, St Louis, MO) 100 μg/ml was used for AGL-1 propagation. The GV3101 strain (background C58; Ti-plasmid cured) was kindly provided by Paolo Costantino (Department of Biology and Biotechnology “La Sapienza” University, Rome, Italy). Rifampicin (25 μg/ml) and gentamicin (25 μg/ml), both provided by Duchefa Biochemie, were used for GV3101 propagation.

### Plasmids

**Figure 1:**
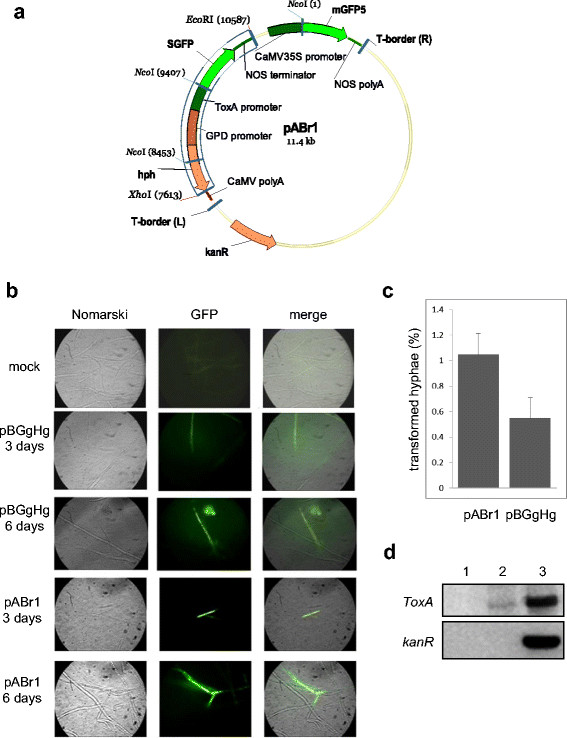
**ATM-transformation with the pABr1 vector. a)** pABr1 (11.4 kb) vector map: *Hygromycin (R),* hygromycin phosphotransferase gene (*hph*); Kanamicin resistance gene (*kanR*); *ToxA promoter,* promoter sequence from *P. tritici-repentis; SGFP,* the S65T variant of the green fluorescent protein (GFP); *CAMV35S* cauliflower mosaic virus promoter; *mGFP5*, folding-enhanced green fluorescent protein variant; *NOS polyA*, Nopaline synthase terminator sequence; *T-border (R)* and *T-border (L)*, right (RB) and left (LB) borders of *A. tumefaciens* T-DNA. The blue highlighted box is the plasmid region derived from pCT74. **b)** Images (100x magnification) of *T. borchii* hyphae transformed with either the pABr1 or the pBGgHg vector, plus an untransformed control (*mock*), obtained by phase-contrast (*Nomarski*) and GFP fluorescence microscopy (*GFP*). The type of treatment, vector and co-cultivation times are indicated on the left; a merge of the Nomarski and GFP images is shown in the rightmost panels. **c)** Quantification of transformed hyphae obtained with pBGgHg or pABr-1, expressed as percentage with respect to the total number of hyphae (~4500) present in each analyzed section. Data are the mean ± s.e.m. of at least five independent experiments. **d)** PCR amplification with *ToxA*-specific primers of total DNA extracted from mock-infected mycelia (*lane 1*), pABr1-transformed mycelia (*lane 2*), and pABr1/AGL-1 bacterial cells (*lane 3*) is shown in the *upper panel*. The results of PCR amplification of the same samples with *kanR*-specific primers are shown in the *lower panel*.

The binary plasmid vector pBGgHg (Chen et al. [2000]) consists of a pCAMBIA 1300 (CAMBIA, Canberra, Australia), backbone containing the *Escherichia coli* hygromycin B phosphotransferase (*hph*) gene and the enhanced green fluorescent proteins (*egfp*) gene, both under control of the strong and constitutive glyceraldehyde-3- phosphate dehydrogenase (*gpd*) promoter from *Agaricus bisporus* and the *CaMV35S* terminator. The plasmid was kindly provided by Peter Romaine (Department of Plant Pathology, Pennsylvania State University). The binary vector pABr1 (Figure 1a) was derived from pCAMBIA 1302 (CAMBIA, Canberra, Australia) through the incorporation of a region extracted from pCT74 (Ciuffetti et al. [1997]; Lorang et al. [2001]). This region contains a synthetic green fluorescent protein (*sgfp)* gene under control of the strong and constitutive *ToxA* promoter from *Pyrenophora tritici-repentis* and a hygromycin B phosphotransferase (*hph*) gene under control of the constitutive promoter *pdgp* from *Agaricus bisporus.* To generate pABr1, both starting plasmids were subjected to *Eco*RI-*Xho*I digestion, which removed the T-DNA region of pCAMBIA 1302 containing the plant *hph* gene under control of the *CaMV35S* promoter, while the *GFP* gene controlled by the *CaMV35S* promoter was retained. The binary plasmid vector pABr3 is a shortened version of pABr1 from which the region containing the *GFP* gene under control of the *CaMV35S* promoter used for plant transformation was deleted.

### ATM transformation

*T. borchii* mycelia were pre-grown in the dark at 24°C for 7 days on cellophane-overlaid PDA plates and then transferred to PDA medium containing 200 μM acetosyringone (AS) to stimulate *Agrobacterium* virulence (outlined in Additional file 1: Figure S1). Three days before the start of co-cultivation, pre-transformed *Agrobacterium* cells were grown at 28°C for 2 days in the presence of the appropriate selection marker antibiotics (100 μg/ml carbenicillin for AGL-1; 25 μg/ml each of rifampicin and gentamycin for GV3101; 30 μg/ml kanamycin for both vectors). On the day of co-cultivation, a fresh overnight bacterial culture with an optical density at 600 nm (OD_600nm_) of 1.2-1.5 was diluted to an OD_600nm_ of 0.075 with antibiotic-supplemented medium also containing 200 μM AS and grown for 4 h to an OD_600nm_ of 0.3. After reaching this cell density, 50 μl of bacteria in LB (1.5 × 10^3^ cells/μl) were overlaid onto *T. borchii* mycelia and co-cultivation was continued for 3 days at 22-24°C. Following co-cultivation, mycelia were washed with sterile ddH_2_O and 400 μM cefotaxime, and mycelial samples were sliced and examined by confocal microscopy. The remaining mycelia were transferred to new plates of Evans minimal medium containing 400 μM cefotaxime (to inhibit bacterial growth) and 15 μg/ml hygromycin (to aid in transformant hyphae isolation). Additional mycelial samples were analyzed after 6 days of co-cultivation, and the remaining mycelia were monitored over time for hygromycin B resistance and GFP expression.

### Fluorescence and confocal microscopy

Sections (3 × 3 mm) of either control, untransformed *T. borchii* mycelia or mycelia co-cultivated with *A. tumefaciens* for 3 days were washed 5 times with distilled water and cefotaxime (400 μM) prior to microscopic analysis. An Axioskop 2 microscope (Carl Zeiss International, Oberkochen, Germany) equipped with a 100X oil immersion objective (Plan Neofluar), a Zeiss filter set (green excitation filter 450–490 HB) and a Zeiss AttoArc2 HB-100 W mercury lamp were used for fluorescence microscopy analysis. Images were captured with a Microcolor camera (RGB-MS-C; CRI, Boston, MA) and processed with Diffraction Micro CCD software and Adobe Photoshop (Adobe System, San Jose, CA). Typical exposure times were 30–60 ms for the SGFP and Nomarski images. Confocal laser scanning microscopy was performed with a Leica microscope equipped with a TCS SP2 Laser (Ar/Kr, Gre/Ne, He/Ne; standard phase contrast plus Normaski contrast). SGFP was excited with a 488-nm laser line and detected at 515-530 nm.

### DNA extraction and PCR analysis

Mycelia to be analyzed by confocal microscopy were washed 5 times with ddH_2_O and 400 μM cefotaxime and then disrupted by freezing in liquid nitrogen. DNA was extracted from frozen mycelia using the DNeasy Plant Minikit (QIAGEN N. V., Hilden, Germany) as per manufacturer’s instructions. The amplification protocols described in (Grimaldi et al. [2005]) were employed for PCR analysis of the aminoglycoside 3'-phosphotransferase (*kanR*) and *sgfp* genes, using the oligonucleotide primers listed below.

*kanR* primers (to amplify a region comprised within the binary plasmid vector):

kanR_Fw: 5′-GGTCATGCATTCTAGGTACT-3′; kanR_Rw: 5′-AATGGCTAAAATGAGAATAT-3′.

*Sgfp* primers (to amplify a region comprised within the T-DNA):

sgfp_Fw: 5′- CACATGAAGCAGCACGACTT-3′; sgfp_Rw: 5′-TGCTCAGGTAGTGGTTGTCG-3′;

*ToxA* promoter region (also comprised within the T-DNA):

ToxA_Fw: 5′-GGGGAATTCTAGTGGAACTGATTGGAATGCA-3′;

ToxA_Rw: 5′-GGGGATAGAACCCATGGCCTATAT-3′

### DNA blot analysis

Genomic DNA from transformed and untransformed (negative control) *T. borchii* mycelia (10 μg each) plus the same amount of pABr1 plasmid DNA (positive control) were digested with *Nco*I. A separate sample of pABr1 was digested in parallel in order to generate a pair of DNA fragments to be used as probes. Four fragments were obtained from *Nco*I-*Xho*I digestion, including an 840 bp fragment that was used as probe. This fragment comprises a region (located around the T-DNA junction site) that encompasses the site of recombination with genomic DNA. The second probe was obtained by *Nco*I digestion, which produced three different fragments. The fragment that was utilized as probe was 954 bp in length (from the middle of the hygromycin phosphotransferase cassette to the *ToxA* promoter). This probe matches the region of the T-DNA that is transferred to *T. borchii* hyphae. Radioactive labeling with approximately 20 ng of purified templates, blotting, hybridization and washing were performed as described (Green and Sambrook [2012]; Montanini et al. [2003]).

### TAIL-PCR amplification, cloning and sequencing of T-DNA integration sites

Genomic DNA sequences flanking the T-DNA were amplified by Thermal Asymmetric Interlaced PCR (TAIL-PCR) as described in (Wang and Li [2008]). The following oligonucleotides were used as primers to amplify the Left Border (LB) and the Right Border (RB) regions, in combination with the degenerate, genome-sequence targeting primer AD1.

LB1 5′-GGGTTCCTATAGGGTTTCGCTCATG-3′ LB2 5′-CATGTGTTGAGCATATAAGAAACCCT-3′ LB3 5′-GAATTAATTCGGCGTTAATTCAGT-3′

RB1 5′-GGCATGGCCGTCGTTTTACAAC-3′ RB2 5′-AACGTCGTGACTGGGAAAACCCT-3′ RB3 5′-CCCTTCCCAACAGTTGCGCA-3′

AD1 5′-WGTGNAGWANCANAGA-3′

DNA amplified by TAIL-PCR was cloned into the pDrive A/T vector (QIAGEN) according to the manufacturer’s instructions, followed by DNA sequence analysis of plasmids isolated from transformant colonies.

### Other procedures

Mycelial sections (3 × 3 mm) containing ~4500 hyphal apexes, as estimated by counting with a Burker chamber, were used for transformation efficiency determinations. The fraction (%) of transformed hyphae was obtained by dividing the number of fluorescent hyphae (average of multiple determinations) by 4500; data were calculated as the mean ± standard error of the mean (s.e.m.) of at least five independent replicates.

The Vector NTI® Software (Life Technologies) was used for drawing plasmid maps. BLAST searches were conducted against the *T. melanosporum* genome sequence at the MycorWeb site (http://mycor.nancy.inra.fr).

## Results

### ATM transformation of *T. borchii* mycelia using a *Pyrenophora ToxA* promoter-bearing binary vector

We previously documented the production of transformed, GFP-expressing *T. borchii* mycelia with the use of ATM and the pBGgHg plasmid (Grimaldi et al. [2005]). However, transformation efficiency was rather low (0.2-0.5%) with a fairly weak GFP signal that was lost after a few weeks, possibly due to the lack of stable integration. In order to improve transformation efficiency and to achieve higher GFP expression levels as well as stable transformants, a new series of cloning vectors for ATM transformation was constructed. We first created a binary vector, named pABr1 (Figure 1a), with the *sgfp* gene under control of the *ToxA* promoter from the ascomycete *P. tritici-repentis* (Ciuffetti et al. [1997]), inserted within the T-DNA region, which also contains the hygromycin phosphotransferase *(hph*) gene utilized for positive selection of transformants. The *sgfp* gene, which codes for the S65T variant of GFP, is a commonly utilized transformation marker in fungi (Niwa [2003]) and use of the *ToxA* promoter has previously been shown to enhance the expression of reporter genes in various fungi including *Neurospora crassa* (Lorang et al. [2001]). Our goal was to couple the enhanced strength of the *ToxA* promoter with the increased stability of SGFP in order to obtain a stronger fluorescence signal and thus an improved visualization of transformed hyphae. To this end, we performed ATM transformation by treating mycelia with the AGL-1 strain, carrying either pABr1 or pBGgHg, and comparing the transformation efficiency of these two vectors. After 3 days of co-cultivation (outlined in Additional file 1: Figure S1), a subset of mycelia was transferred to new antibiotic-containing plates (ampicillin, cefotaxime and hygromycin B) and samples were taken for hyphal section visualization by confocal microscopy. This allowed the identification of fluorescent, GFP-producing hyphae, by comparison with the background signal associated with control mycelia transformed with the empty AGL-1 strain (Figure 1b). The diffuse fluorescence background present in mock-transformed samples, contrasted with the localized signal observed in the apical hyphae of *T. borchii* transformed with AGL-1 carrying the binary vector. Transformation efficiency obtained with the pABr1 vector after 3 days of co-cultivation was consistently found to be at least two-fold higher (≥1%) than that obtained with pBGgHg using the same AGL-1 *A. tumefaciens* strain (Figure 1c). This was calculated as the ratio between fluorescent hyphae and the total number of hyphae per section (~4500). When the analysis was repeated at 6 days, i.e. after 3 additional days since the end of co-cultivation, the hyphae appeared more fluorescent, likely due to SGFP accumulation (Figure 1b), but without any appreciable change in the relative number of fluorescent hyphae (data not shown). Still, the SGFP signal in pABr1-transformed hyphae appeared stronger than that of hyphae transformed with the pBGgHg vector, thus facilitating the identification of transformants grown over time. Transformation was also verified by PCR amplification of the *ToxA* promoter using genomic-DNA extracted from transformed mycelia as template (Figure 1d). To confirm that *ToxA* amplification did indeed result from the mobilized T-DNA rather than from residual bacteria, we performed a parallel PCR analysis using a pair of primers annealing to the kanamycin resistance (*kanR*) gene region that is present in the plasmid outside of the T-DNA. The absence of a *kanR* amplification signal ruled out a false positive result caused by residual contaminating bacteria. We then used a different *Agrobacterium* strain (GV3101) to verify that the improved transformation efficiency obtained with pABr1 was a genuine effect of the plasmid vector, rather than an indirect effect of the specific *A. tumefaciens* strain utilized for transformation. The results obtained with GV3101 confirmed that also in this strain the transformation efficiency yielded by pABr1 was at least two-fold higher than that obtained with pBGgHg (Additional file 1: Figure S2a,b). However, global efficiency with GV3101-mediated transformation was generally lower than that obtained with AGL-1 (Additional file 1: Figure S2c), suggesting that not all *A. tumefaciens* strains are equally effective for *Tuber* transformation. Also in this case, the data were confirmed by PCR analysis, which revealed an *sgfp* amplicon in the case of DNA extracted from transformed mycelia*,* but not in the case of mock-infected mycelia (Additional file 1: Figure S2d). Furthermore, a stronger GFP signal was systematically observed in pABr1 transformants compared to transformants obtained with the pBGgHg vector utilized in previous studies (Grimaldi et al. [2005]). Altogether the data confirm the superior performance of the newly designed pABr1 vector.

### T-DNA transformation occurs by random integration in the *Tuber* genome

**Figure 2:**
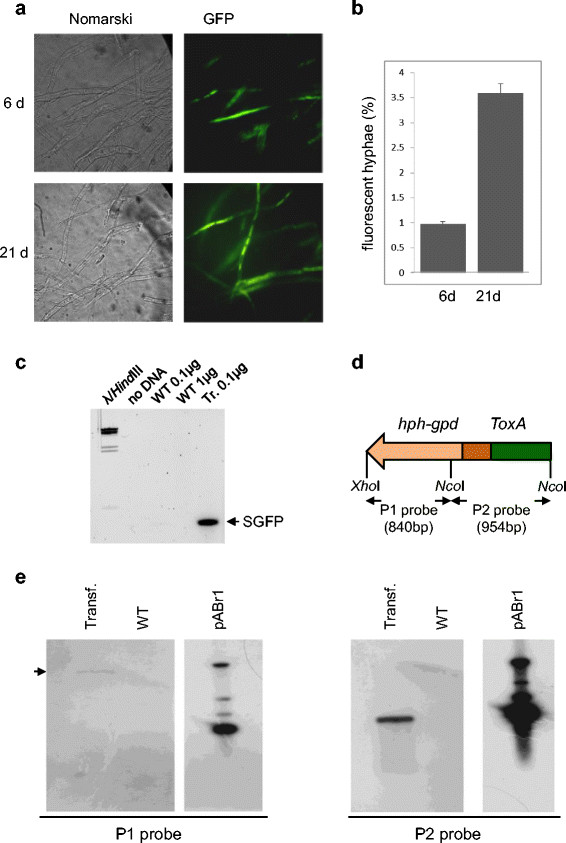
**Following the fate of the T-DNA in pABr1-transformed hyphae. a)** Sections (3 x 3 mm) of transformed *T. borchii* mycelia analyzed by confocal microscopy (GFP wavelength) at the indicated times after *Agrobacterium* infection. **b)** Histogram representation of cumulative data produced by the experiments in panel **a)** expressed as percentage of fluorescent hyphae with respect to the total number of hyphae present in each section (~4500). Data are the mean ± s.e.m. of at least five independent experiments. **c)** PCR amplification with *sgfp* specific-primers of genomic DNA obtained from mock-transformed mycelia (*WT*) using 0.1 μg (*lane 3*) and 1 μg (*lane 4*) of template DNA, and from AGL-1/pABr1-transformed mycelia (0.1 μg of template DNA; *lane5*). A PCR negative control (*no DNA*) and size markers (*λ/HIII*) are shown in the two leftmost lanes. **d)** Schematic representation of the probes (P1 and P2) utilized for DNA blot analysis. **e)** DNA blot analysis of DNA extracted from AGL-1/pABr1-transformed mycelia (“*transformant*”, *lane 1*), mock-transformed mycelia (*WT*, lane 2), and AGL-1/ABr-1 bacterial cells (*pABr1*, lane 3), hybridized with the P1 probe, which detects T-DNA integration regions. After stripping, the same membrane was hybridized with the P2 control probe.

As described previously (Bundock and Hooykaas [1996]), the T-DNA can either be integrated into the host genome or transferred and incorporated as stable, extra-chromosomal T-DNA elements that can form circular structures in plants (Singer et al. [2012]). To investigate this point, we used a phenotype-based approach. We hypothesized that if the T-DNA was integrated in the *Tuber* genome, the same transformant section analyzed at different times after transformation should show a progressive increase in the fluorescence of neighboring hyphae, resulting from multiple nuclear divisions of hyphae containing the integrated transgene. We thus isolated regions of AGL-1/pABr1-transformed mycelia containing one or very few transformed hyphae (visualized by confocal microscopy after 6 days of co-cultivation; see Figure 2a “6d”) and, after sterile washing, put them back to liquid medium for an additional 15 days. Subsequent analysis by confocal microscopy revealed a four-fold increase in the number of contiguous fluorescent hyphae (see Figure 2a “21d” and b), in keeping with the hypothesis that transformation mainly occurs via stable integration rather than through the incorporation of extra-chromosomal elements. Transformation was confirmed at the molecular level by the detection of an *sgfp* PCR amplification product in transformed mycelia and the lack of a similar product when using increasing amounts of genomic DNA from mock infected wild-type mycelia as template (Figure 2c). To demonstrate the presence of integrated transgene copies, we further investigated the fate of the transgene by DNA blot analysis. Since very little amounts of genomic DNA can usually be obtained from *Tuber* mycelia, and the number of transformed nuclei is far less than that of untransformed nuclei, unique regions of integration were difficult to visualize. To overcome this problem, also considering that random, non-sequence specific T-DNA integration has been shown to be the main outcome of ATM-transformation in other fungi (Bundock et al. [2002]), we mapped T- DNA insertions at the genomic level by hybridization analysis conducted with two specific probes (named P1 and P2).

The P1 probe was obtained by using an 840 bp sequence derived from *Nco*I-*Xho*I digestion of pABr1 as template (Figure 2d). P1 recognizes the T-DNA region between the left border and the *hph* sequence. This region presumably recombines with the *Tuber* genome as described in other fungi (Thomas and Jones [2007]). The other probe (P2) was generated by using as template, a 954 bp region of the T-DNA obtained by *Nco*I digestion (Figure 2d). This probe recognizes an internal sequence comprising part of the *hph* gene, the *pdgp* promoter and the entire *ToxA* promoter sequence, that is present regardless of the integrated or extra-chromosomal state of the T-DNA. The P2 probe was thus used as a positive control for hybridization experiments, to verify that DNA blotting had been performed correctly. Total genomic DNA from transformed and untransformed mycelia was then subjected to *Nco*I digestion, which would generate a smear of fragments recognized by the P1 probe only in the case of T-DNA integration. As shown in Figure 2e (left panel), a weak, P1 probe-positive band was only observed in transformed mycelia. The fact that the size of this band (~10,000 bp) was much higher than expected (840 bp) strongly suggests the occurrence of an integration event. In the same sample, a stronger signal resulting from the sum of integrated and extra chromosomal T-DNA fragments of the same size, was observed with the P2-probe, which hybridized with a DNA fragment, located within the T-DNA region, having an expected size of 954 bp (Figure 2e, right panel). Also in this case, the P2-associated signal was only observed in the case of DNA derived from transformed, but not mock-infected, mycelia. Altogether these data suggest the integration of at least some copies of the pABr1-derived T-DNA in the *Tuber* genome.

### Improved transformation efficiency of the shortened pABr3 vector

**Figure 3:**
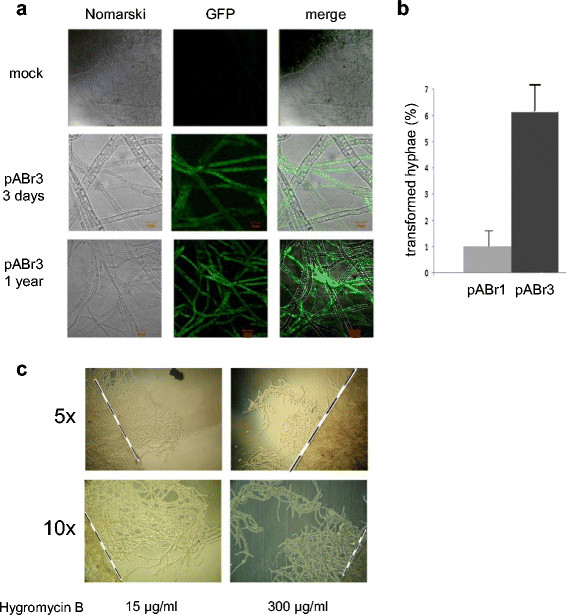
**pABr3 transformation, comparison of transformation efficiency with pABr1, and hygromycin transformant amplification. a)** Images (100x magnification) of pABr3-transformed hyphae, at 3 days and 1 year after transformation as indicated, obtained by phase-contrast (*Nomarski*) and SGFP fluorescence microscopy. A negative, untransformed control (*mock*) is shown at the top; a merge of the two types of images is shown in the rightmost panels. **b)** Comparison of the transformation efficiencies obtained with pABr1 and pABr3. Data are the mean ± s.e.m. of at least five independent experiments. **c)** Transformed mycelia were grown on hygromycin (15 μg/ml)-supplemented medium for 60 days and then transferred to solid medium containing the indicated concentrations of hygromycin B. After 7 days, mycelia were visualized under a stereo-microscope at the indicated magnifications to detect the presence of tufts grown beyond the dotted line.

pABr1 is a binary vector containing the T-DNA sequence interposed between the left border and the right border. It also contains an unmodified *gfp* gene, under control of the *CaMV35S* promoter and a *NOS* terminator (necessary for plant transformation), between the *Hin*dIII site and the right border of the T-DNA (Figure 1a). While contributing to, and considerably expanding the size of the T-DNA, this region is presumably not necessary for *Tuber* transformation and it was thus eliminated. The T-DNA of the resulting vector, designated as pABr3, only contained sequences derived from the pCT74 plasmid (*hph* gene*, gpd* promoter, *ToxA* promoter and the *sgfp* reporter gene). We hypothesized that the smaller size of the T-DNA sequence harbored by pABr3, corresponding to approximately 1/3 of the sequence contained in pABr1, may result in a higher transformation efficiency, thus increasing the number of transferred T-DNA copies. Indeed, as shown in Figure 3, a comparison of pABr1- and pABr3-transformed hyphae (obtained after 3 days of co-cultivation with the AGL-1/pABr1 and AGL-1/pABr3 *Agrobacterium* strains) revealed an approximately 6-fold higher transformation efficiency with the latter binary vector, likely resulting from the combined effect of a smaller sized T-DNA and an enhanced expression of the SGFP reporter. Moreover, as revealed by confocal microscopy, the SGFP signal was found to be strikingly stable and diffuse even 1 year after the initial transformation, further indicating the increased performance of this optimized-transformation procedure.

Since it was previously impossible to obtain portions of *Tuber* mycelia capable of growing on media containing more than 15 μg/ml hygromycin B, we also analyzed the ability of AGL-1/pABr3-transformed mycelia to grow in the presence of increasing amounts of hygromycin B. To this end, transformed mycelia were transferred to plates containing 15 μg/ml hygromycin B and the outline of the mycelium was marked so to allow an easy visualization of mycelial clumps overgrowth over time. After 60 days (during which mycelia were transferred to fresh antibiotic-containing medium every 10 days), full-bodied mycelial clumps were found to propagate on medium containing 15 μg/ml of hygromycin B (Figure 3c, top panel). Following isolation and transfer of hygromycin-resistant mycelial slices to media containing increasing concentrations of hygromycin B, we found that transformants were able to propagate in the presence of hygromycin B concentrations as high as 300 μg/ml (Figure 3c, bottom panel). Mycelia subjected to this high-stringency selection procedure were used as starting material for further molecular analyses (see below).

### Integration site identification by TAIL-PCR

**Figure 4:**
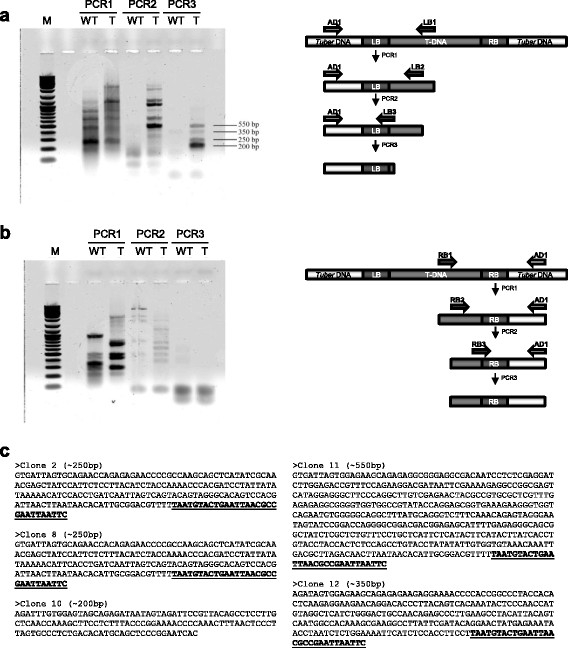
**TAIL-PCR and integration site identification. a)** TAIL-PCR amplification of DNA fragments covering pABr3 integration sites. Representative results obtained from three amplification runs (PCR1, PCR2, PCR3) carried out with three nested primers annealing with the left border (LB) of the T-DNA (LB1, LB2 and LB3) and a degenerate primer annealing with the neighboring genome sequence (AD1) (see explanatory scheme on the right and “Materials and Methods” for details). Template genomic DNA was extracted from a pABr3-transformed mycelium (*T*) and from a mock-transformed mycelium (*WT*); size markers (*M*) are in the leftmost lane. **b)** Amplification of the right border (RB). **c)** Integration site sequences retrieved from TAIL-PCR analysis of transformants #2, #8, #10, #11 and #12; The underlined and highlighted in bold sequences represent the portion of the T-DNA adjacent to the unknown sequence obtained by PCR.

The above results suggest that *A. tumefaciens* transforms *T. borchii* via genomic integration of the T-DNA. We further investigated this point by isolating T-DNA integration sites comprised of unknown *Tuber* genome sequences flanking known sequences of the T-DNA borders. To overcome potential difficulties deriving from the syncytium organization of *Tuber* hyphae, which upon transformation become a mosaic with only one transformed nucleus surrounded by a multitude of untransformed nuclei, we used TAIL-PCR as a sensitive tool to recover flanking-DNA sequences (Liu and Whittier [1995]). This consisted of three subsequent PCR cycles amplifying a region interposed between the T-DNA border and the flanking genomic DNA using different, LB or RB, site-specific primers and a degenerate primer. The high specificity of this reaction makes TAIL-PCR a powerful tool to verify T-DNA integration in fungal cells after ATM transformation (Rolland et al. [2003]). DNA obtained from mycelia transformed with AGL-1/pABr3 and subjected to high-stringency hygromycin selection, together with DNA from control untransformed mycelia was used for TAIL-PCR analysis. While the first cycle of TAIL-PCR, conducted on the left border, generated non-specific amplification products, the last cycle yielded specific products that were only detectable in genomic DNA from transformed mycelia (Figure 4a). The same approach applied to the right border did not yield any amplicon (Figure 4b). This is a common problem previously described in various plant studies (Mazars et al. [1991]) and it is likely caused by illegitimate recombination events that also occur during fungal transformation (Choi et al. [2007]). Sequence analysis of transformant-specific LB amplification products revealed T-DNA/genomic DNA junction sites (Figure 4c). The non-T-DNA portions of the sequences thus retrieved were used as queries for a BLAST search performed against the heterologous, but likely closely related, *T. melanosporum* genome (Martin et al. [2010]). Two sequences (#10 and # 11) yielded positive matches, corresponding, respectively, to a region between two closely located transposable elements and to a retrotransposable element gene. The apparent lack of positive matches for the other non-T-DNA sequences retrieved from TAIL-PCR may be explained by the relatively short length of such sequences compared to the extremely high number and variability of transposons and other repeated elements in the *T. melanosporum* genome utilized as reference for BLAST search. TAIL-PCR analysis thus provided final proof of the integrative transfer of the T-DNA region into the *Tuber* genome.

## Discussion

*Tuber* spp. are ecologically and economically important filamentous Ascomycetes. These fungi, whose fruitbodies are commonly known as truffles (Pegler et al. [1993]), have a subterranean habitat and establish a symbiotic relationship with several trees and shrubs via ectomycorrhiza formation (Hebe et al. [1999]). Crucial for the success of the ectomycorrhizal interaction is the mutualistic exchange of nutrients between the symbiont and the host plant. Indeed, inoculation of plants with *Tuber* mycelia leads to an increased plant growth, indicating that the fungus is a mutualistic symbiont (Giovannetti and Fontana [1982]). Recently, the genome sequence of a representative *Tuber* species, the black truffle *T.melanosporum*, has been determined (Martin et al. [2010]). Although a large fraction of genes has been functionally defined by sequence homology, a consistent number of them (>40%) remains functionally unknown. Reverse genetic analysis will thus be required in order to assign a role to these orphan genes. In *Tuber*, conventional genetic transformation procedures failed due to inefficient protoplast regeneration (Poma et al. [2006]). We sought to circumvent this problem with the use of *A. tumefaciens-*mediated transformation, a foreign DNA transfer strategy largely used in plants (Sheng and Citovsky [1996]) and fungi (Michielse et al. [2005]).

Our first attempt toward ATM-transformation in *T. borchii* was based on the pBGgHg binary vector (Grimaldi et al. [2005]). Although the T-DNA entered host cells, transformation was transient and the organization of T-DNA (extrachromosomal vs. integrated) was not clear. Moreover, transformation efficiency was rather low (0.2-0.5%) and the EGFP fluorescence signal as well as hygromycin resistance were lost quite early, typically after 4–5 weeks of in vitro culture. To overcome these limitations, while trying to achieve integrative transformation, we built two new binary vectors (pABr1 and pABr3). In both pABr1 and pABr3, GFP expression is driven by the strong *ToxA* promoter from the pathogenic fungus *Phirenophora trittici repentis,* which has been employed successfully for transgene overexpression in other filamentous fungi (Freitag et al. [2001]; Lorang et al. [2001]). Transformation with AGL-1/pABr1 (Figure 1a) produced stable transformants with a strong and long-lasting SGFP expression (Figure 1b). Also, transformation efficiency increased by about two-fold compared to AGL-1/pBGgHg (Figure 1c). This difference in transformation efficiency was confirmed with GV3101, an *Agrobacterium* strain less virulent than AGL-1 (Additional file 1: Figure S2).

A further increase in transformation efficiency (~6-fold compared to pBGgHg) was obtained with pABr3 (Figure 3a, b), a shortened derivative of pABr1, lacking the unnecessary plant promoter/GFP/NOS terminator region (Figure 1a). This suggests that a smaller T-DNA size facilitates DNA transfer into host cells. Another important goal in order to strengthen selection efficiency was to maximize the concentration of hygromycin B that could be tolerated by transformed mycelia. Unlike transformation with the other AGL-1/binary vector combinations, AGL-1/pABr3 allowed the amplification of transformed mycelial clumps capable of growing in the presence of hygromycin B concentrations as high as 300 μg/ml (Figure 3c). This enabled the selective isolation and propagation of transformed mycelial regions. This is important, especially considering the lack of conidia-like elements in *Tuber* spp., the syncytial nature of *Tuber* hyphae, and thus the fact that transformants are mosaics, likely bearing more than one transformed locus. We noted, however, that transformed hyphae are not randomly distributed within mycelia, but appear to be concentrated at the periphery of mycelial clumps, the only region from which the fungus could be propagated. This suggests that T-DNA may require a metabolically active tissue for activation and efficient functioning.

With pAbr3, the SGFP signal not only increased in intensity and distribution, but most of the analyzed mycelial sections appeared to be enriched in transformed hyphae even after 1 year from the initial transformation. In fact, in transformed mycelia, the GFP signal progressively became more diffuse and increasingly spread to contiguous hyphae (Figure 3a). This can be explained if the T-DNA is integrated in the *Tuber* genome, so that following duplication of a transformed nucleus the amount of produced/accumulated GFP gradually increases over time.

Integrative transfer was indeed demonstrated by PCR amplification of the *ToxA* (Figure 1d) and the *sgfp* (Additional file 1: Figure S2d) sequences, as opposed to the negative amplification results obtained with the *kanR* gene region, which is located outside of the T-DNA (Figure 1d and Additional file 1: Figure S2d). This was further corroborated by the results of hybridization analyses conducted with region-specific probes. Most notably, the fact that the size of the fragment recognized by a probe hybridizing with the left T-DNA border (i.e., a region that is usually involved in the integration process (Thomas and Jones [2007])) increased from an expected size of 854 bp to ~10,000 bp.

Lastly, we used transformed, hygromycin B-selected mycelia for the identification of the genomic sites of integration by TAIL-PCR (Liu and Whittier [1995]), a widely used approach for the analysis of transformants in systems where homokaryotic colonies are hard to obtain (Poma et al. [2006]). We sequenced T-DNA/*Tuber* genomic DNA flanking site amplicons obtained from transformed mycelia and found that two of them matched transposon-related elements (Figure 4). In *Tuber*, repeated elements, including retrotransposons, account for about 60% of the genome (Martin et al. [2010]), suggesting that integrative transformation mediated by heterologous recombination is likely driven by repetitive DNA sequences.

The highly improved genetic transformation procedure described in this work represents a first, but crucial step toward a functional genomic analysis of *Tuber*, and perhaps other transformation-recalcitrant filamentous fungi as well, with the ultimate goal of producing a comprehensive site-specific mutant collection as in *Aspergillus nidulans*, *N. crassa* and *Magnaporthe oryzae* (http://www.fgsc.net/). Although whole genome sequence data are only available for *T. melanosporum* so far, we used *T. borchii* as an experimental organism to set up this improved transformation procedure because of the easier and more efficient propagation of its mycelium under axenic culture conditions. However, ongoing work, including the setting up of more efficient mycelial growth conditions, indicates that the optimized AGL-1/pABr3-protocol here applied to *T. borchii* also works well in *T. melanosporum*, with the production of well detectable levels of GFP and resistance to hygromycin concentrations as high as 50 μg/ml (A.B and P.B., unpublished).

The next step will be the disruption of non-homologous end joining (NHEJ) mediated by the Ku70 and Ku80 proteins in order to favor integration at homologous sites. The NHEJ pathway, discovered for the first time in mammals (Jeggo et al. [1991]) and so named because it does not require homologous sequences to join DNA ends, repairs double-strand breaks in the absence of a homologous donor (Moore and Haber [1996]). As shown in *N. crassa* (Ninomiya et al. [2004]), disruption of the ku70/ku80 genes strongly enhances homologous recombination and thus the production of site-specific mutants. The presence of ku70/ku80 homologs in the *T. melanosporum* genome (GSTUMT00005220001 and GSTUMT00001928001 gene models; http://mycor.nancy.inra.fr) suggests that such an approach may also be feasible in *Tuber*.

## Abbreviations

ATM: *Agrobacterium tumefaciens*-mediated: 

egfp: Enhanced green fluorescent protein: 

gpd: Glyceraldehyde-3-phosphate dehydrogenase: 

hph: Hygromycin B phosphotransferase: 

kanR: Kanamycin resistance gene, aminoglycoside 3'-phosphotransferase: 

LB: Left border: 

PDA: Potato-dextrose agar: 

RB: Right border: 

sgfp: Synthetic green fluorescent protein: 

TAIL-PCR: Thermal Asymmetric Interlaced PCR: 

T-DNA: Transfer DNA: 

Ti: Tumor-inducing: 

## Competing interests

The authors declare that they have no competing interests.

## Authors’ contributions

AB was in charge of plasmid vector construction and confocal microscopy analysis, set up the transformation protocol and prepared the first draft of the manuscript; BM contributed to plasmid vector construction, confocal microscopy analysis, final editing of the manuscript and figure assembly; EM performed Tail-PCR experiments; MP was in charge of integration site analyses and T. melanosporum experiments; PF contributed to data analysis; SO proposed the project, designed part of the experiments and wrote the last draft of the manuscript; PB coordinated the project, designed part of the experiments and contributed to manuscript drafting.

## Additional file

## Supplementary Material

Additional file 1: Figure S1. Outline of the ATM-transformation protocol; **Figure S2.** ATM-transformation with the pABr1 vector using GV3101 as recipient strain. Click here for file
