# Supplementary material for: Integrative gene transfer in the truffle Tuber borchii by Agrobacterium tumefaciens-mediated transformation
Source: AMB Express. 2014 May 29;4:43. doi: 10.1186/s13568-014-0043-x (PMC4052689; doi:10.1186/s13568-014-0043-x)
Supplement: Additional file 1: Figure S1. — Outline of the ATM-transformation protocol; Figure S2. ATM-transformation with the pABr1 vector using GV3101 as recipient strain. [file s13568-014-0043-x-S1.docx]

***AMB Express - Electronic Supplementary Material***

**Integrative gene transfer in the truffle *Tuber borchii* by *Agrobacterium tumefaciens*-mediated transformation**

A. Brenna^1^, B. Montanini^2^, E. Muggiano^1^, M. Proietto^1^, P. Filetici^3^, S. Ottonello^2^ and P. Ballario^1,3,*^

^1^ Pasteur Cenci Bolognetti Foundation, c/o Department of Biology and Biotechnology “Charles Darwin”, La Sapienza University, 00185 Rome, Italy

^2^ Department of Life Sciences, Biochemistry and Molecular Biology Unit, Laboratory of Functional Genomics and Protein Engineering, University of Parma, 43124 Parma, Italy

^3^ Institute of Biology and Molecular Pathology, CNR, 00185 Rome, Italy

^*^ To whom correspondence should be addressed:

La Sapienza University, Piaz.le A. Moro 5, 00185 Rome, Italy

Tel.: +39 06 49912392; Fax: +39 06 4440812

E-mail: paol[a.ballario@uniroma1.i](mailto:ballario@uniroma1.it)t


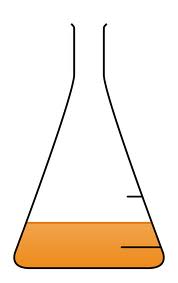

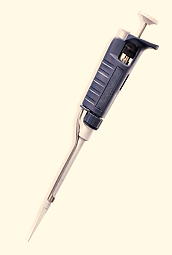

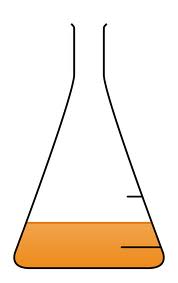

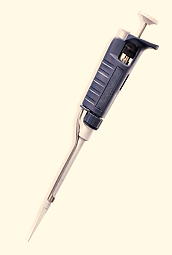


**1**

**2**

**4**

**3**

**5**

7days

200 µM AS

200 µM AS

o/n

4h

3days

15-days old *T. borchii* mycelium transferred to a cellophane membrane-covered PDA plate

Single colony of transformed

*A. tumefaciens* inoculated at 28°C with shaking in LB + antibiotics

Mycelium transferred to a new PDA + 200 µM AS plate

Dilution of *A. tumefaciens* culture into fresh medium (OD_600nm_=0.075) and incubation for 4h at 28°C in LB + antibiotics + 200 uM AS

50 µl of the bacterial culture from step (4) are added to the mycelium and co-cultivation is carried out for 3 days at 22-25°C in the dark

**Figure S1.** **Outline of the ATM-transformation protocol**


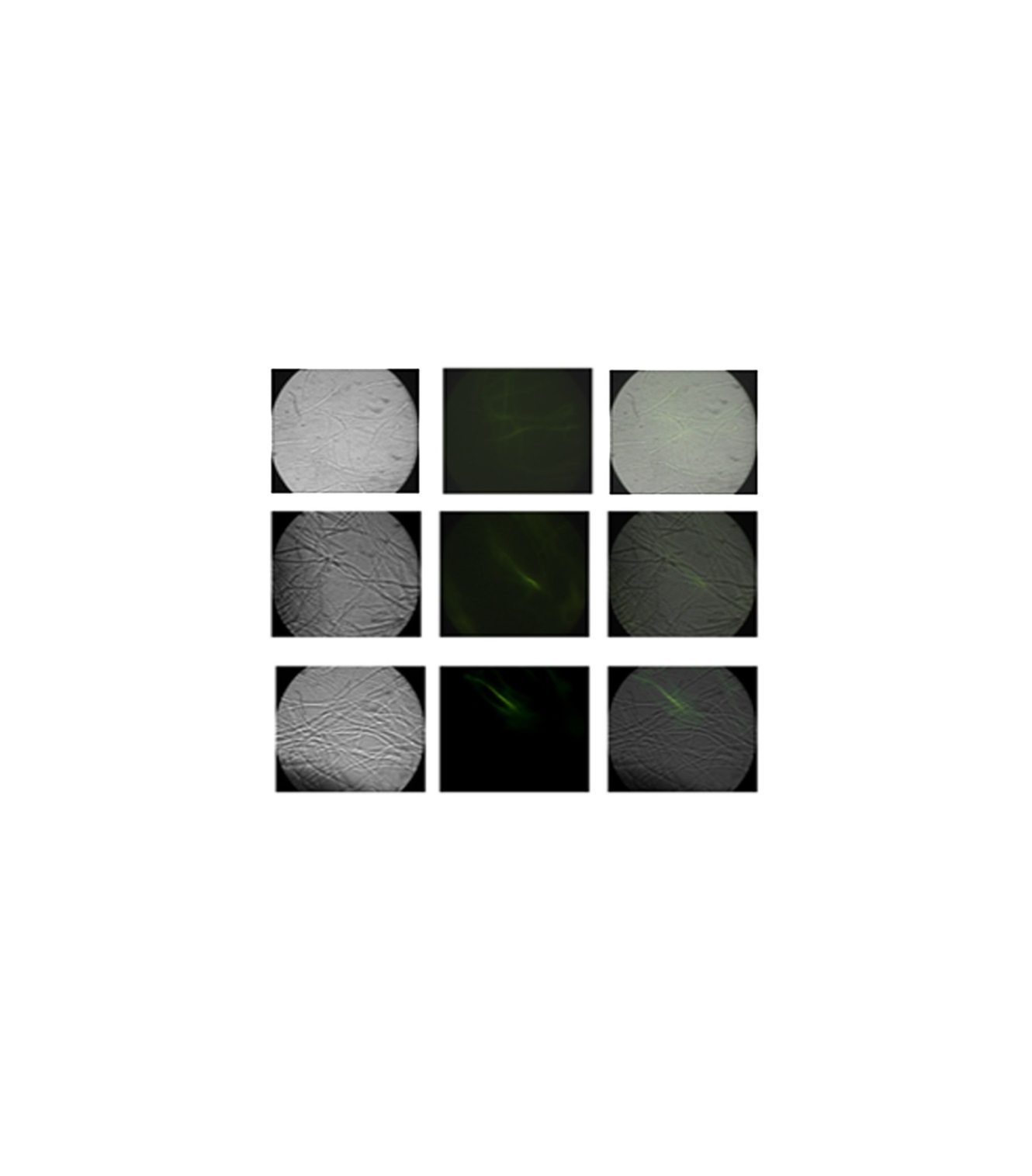

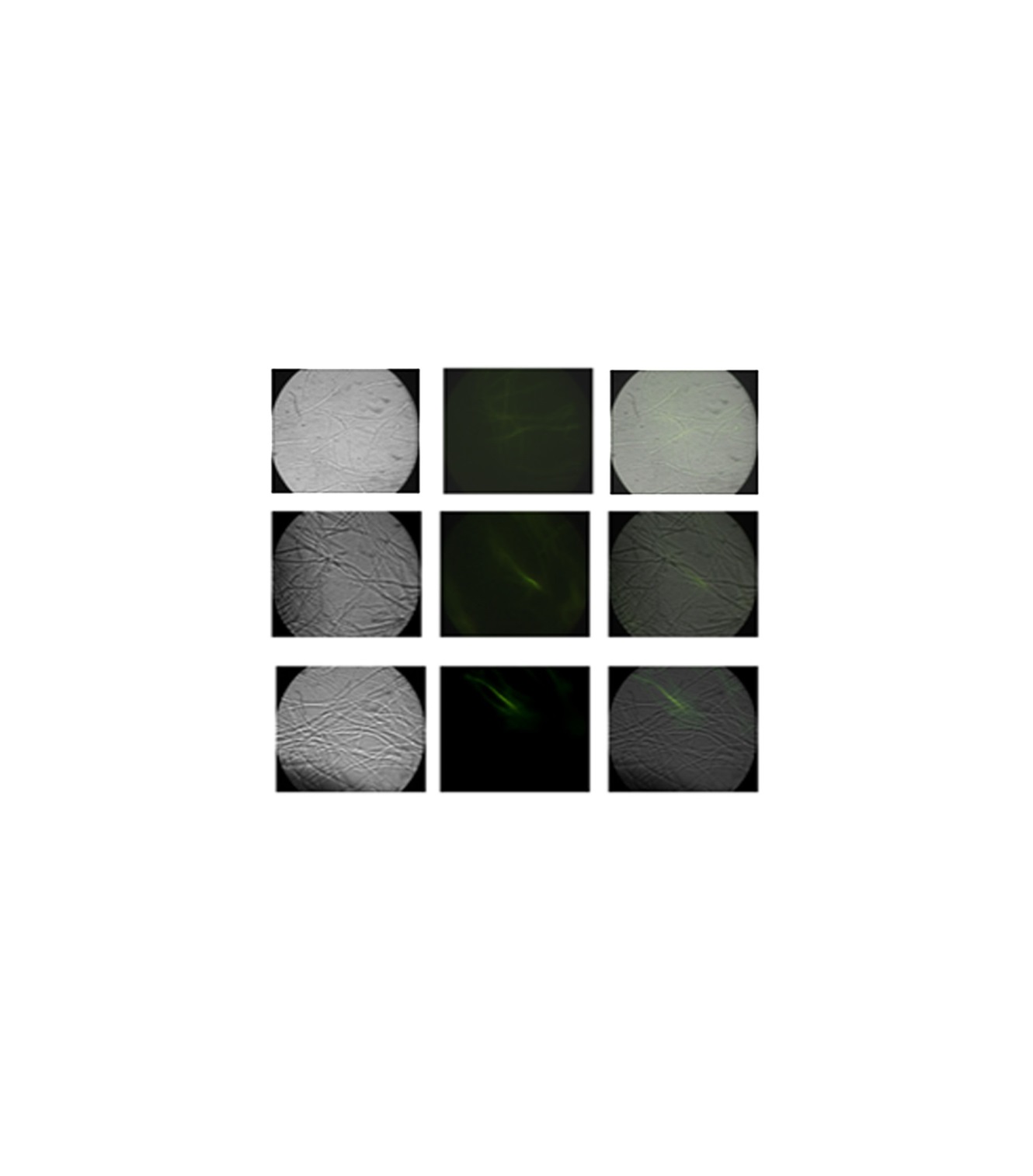

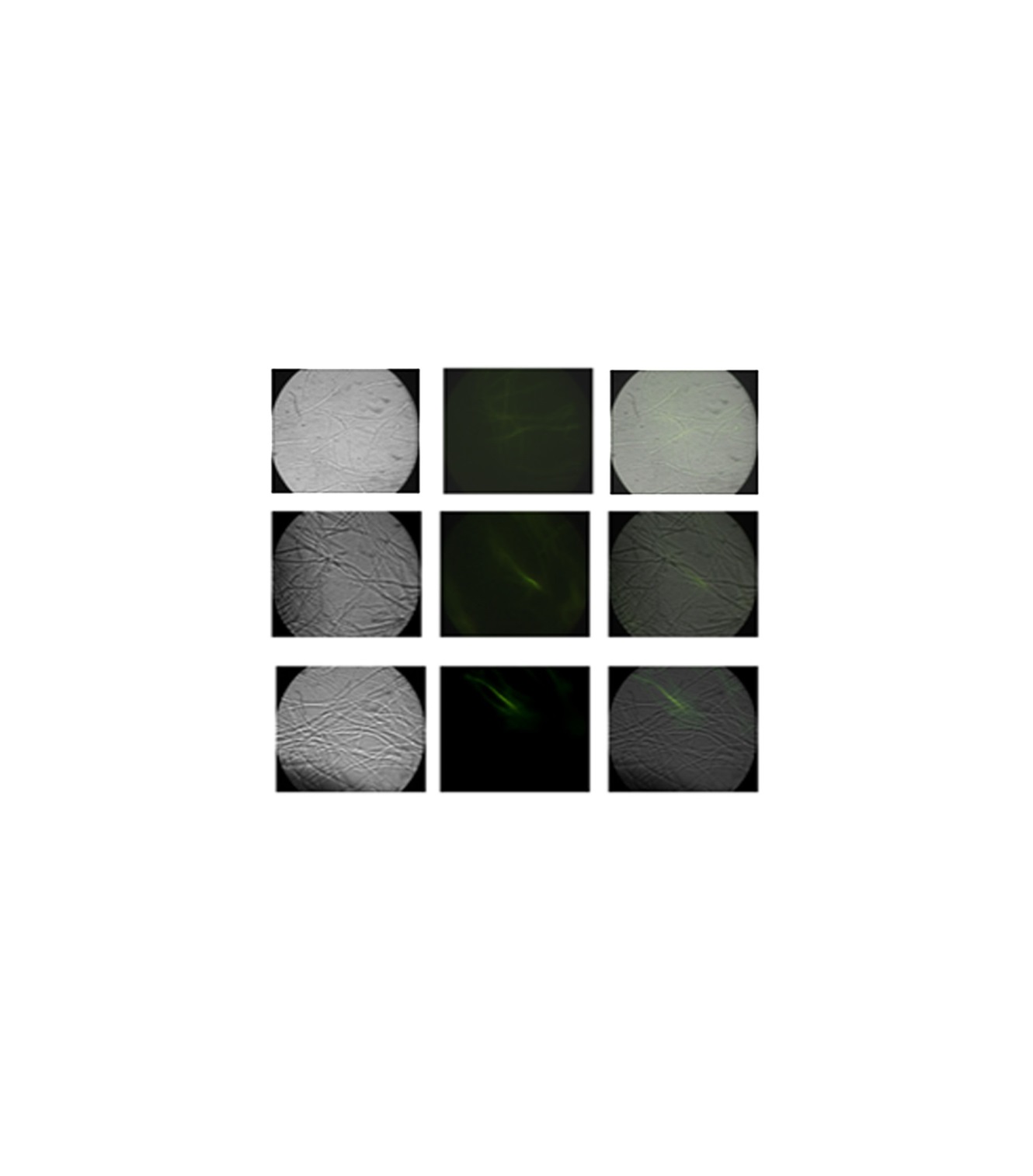

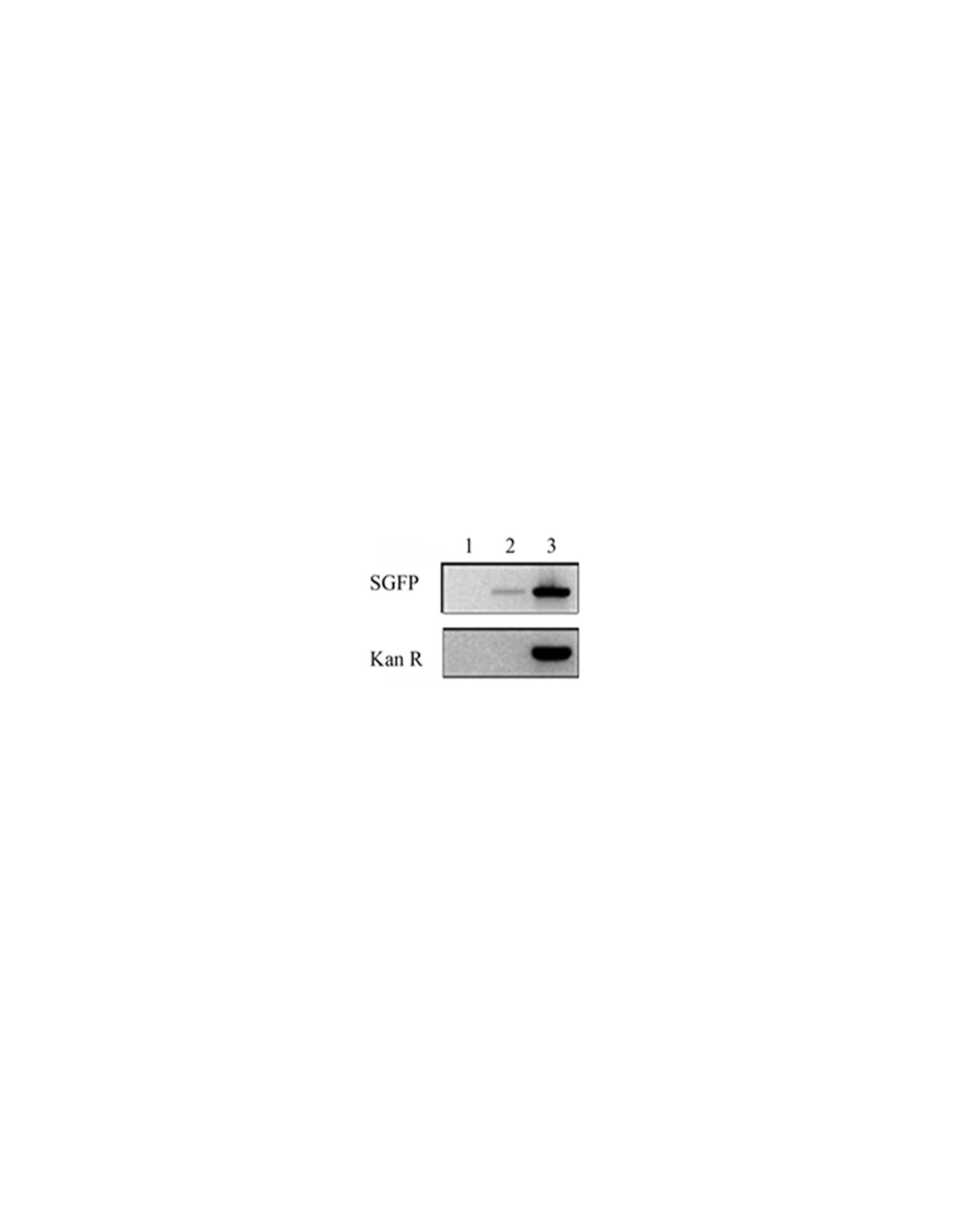


transformed hyphae (%)

pBGgHg

pABr1

**a**

**b**

**c**

**d**

% of transformed hyphae

GV3101

AGL-1

*sgfp*

*kanR*

1 2 3

Nomarski

GFP

merge

mock

pABr1 3 days

pABr1 6 days


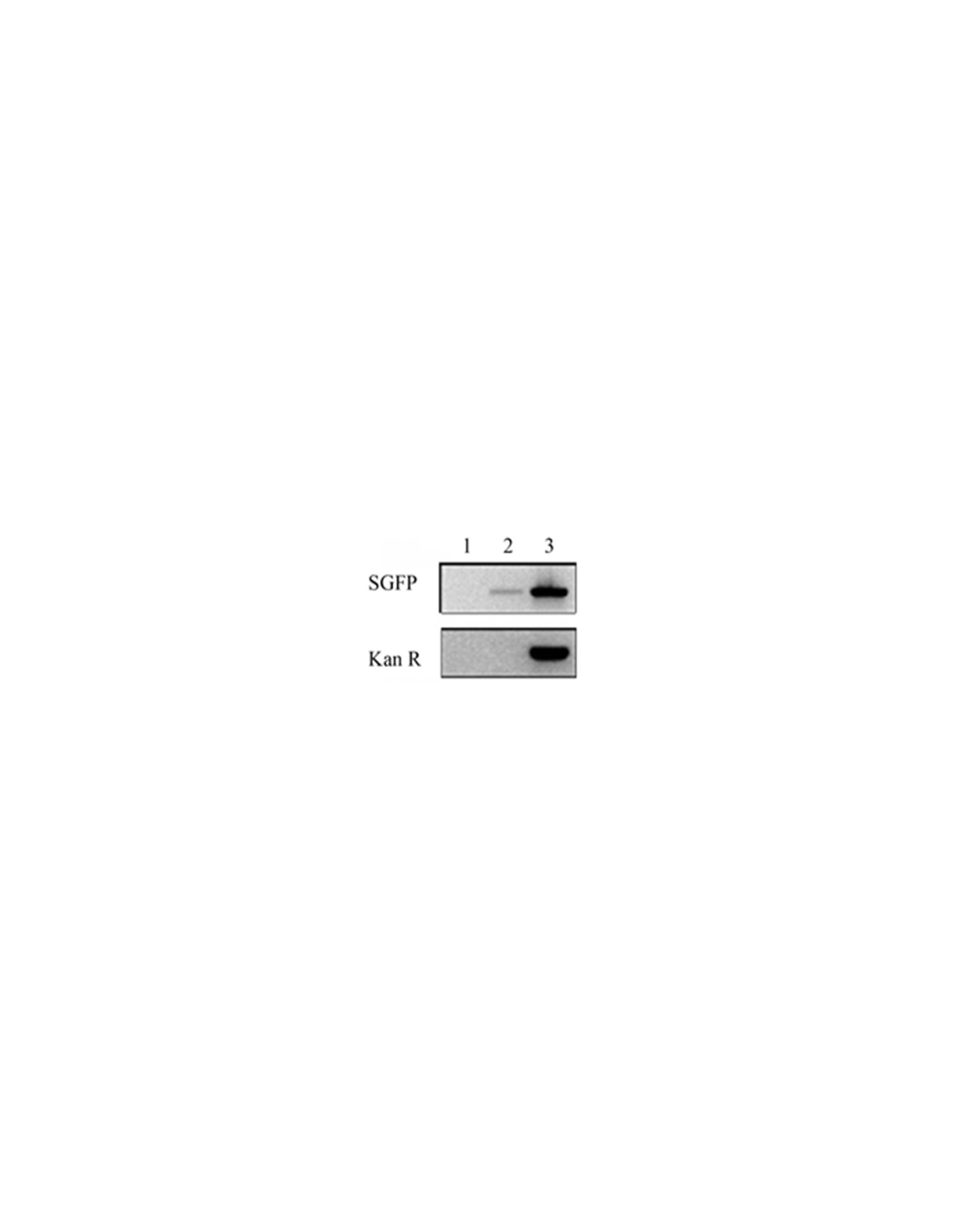

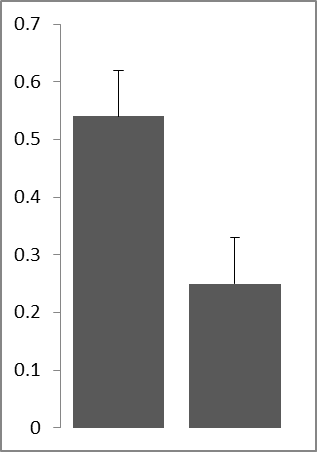

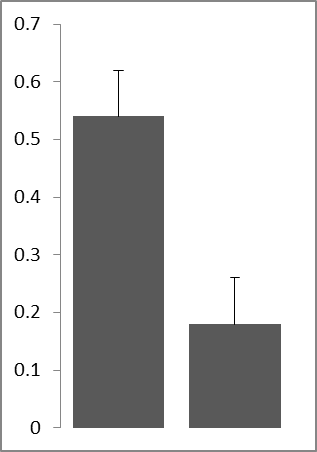


**Figure S2.** **ATM-transformation with the pABr1 vector using the recipient strain GV3101 a)** Images (100x magnification) of *T. borchii* hyphae transformed with the pABr1vector obtained by phase-contrast (*Nomarski*) and *GFP* fluorescence microscopy. Untransformed, control hyphae are shown at the top (*mock*). The type of treatment, vector and co-cultivation times are indicated on the left; a merge of the Nomarski and GFP images is shown in the rightmost panels. **b)** Quantification of transformed hyphae obtained with pABr1/GV3101 and pBGgHg/GV3101 expressed as percentage of fluorescent hyphae with respect to the total number of hyphae (~4500) present in the analyzed sections. Data are the mean ± s.e.m. of at least five independent experiments. **c)** Same as B for the comparison of pABr1/AGL-1 and pABr-1/GV3101. **d)** PCR-amplification of DNA obtained from mock-infected mycelia (*lane 1*), AGL-1/pABr1-transformed mycelia (*lane 2*), and pABr1/AGL-1 bacterial cells (*lane 3*) performed with *sgfp*- (*top*) and *kanR*- (*bottom*) specific primers
